# Antidepressant-like effects of cannabidiol when combined with temozolomide in adult rats

**DOI:** 10.3389/fphar.2026.1873308

**Published:** 2026-06-29

**Authors:** Laura Gálvez-Melero, Sandra Ledesma-Corvi, Cristian Bis-Humbert, M. Julia García-Fuster

**Affiliations:** 1 University Research Institute of Health Sciences (IUNICS), University of the Balearic Islands, Palma, Spain; 2 Health Research Institute of the Balearic Islands (IdISBa), Palma, Spain; 3 Department of Medicine, University of the Balearic Islands, Palma, Spain

**Keywords:** antidepressants, comorbid disorders, depression, drug interactions, glioblastoma

## Abstract

There is a need for characterizing more efficacious therapeutic strategies for glioblastoma multiforme to overcome treatment resistance to the standardized use of temozolomide as well as to better treat its debilitating comorbidities like depression. In this context, combination therapies, particularly cannabidiol with temozolomide exhibited additive or synergistic anti-tumor effects, capable of improving temozolomide’s resistance over the course of treatment. Beyond its antitumoral properties and given the promising antidepressant-like profile of cannabidiol, we aimed at evaluating the pharmacological interaction of a combined therapy with cannabidiol and temozolomide as a potential novel antidepressant-like strategy, in comparison with temozolomide’s combination with fluoxetine, the goal treatment standard for depressive-like symptomatology. To do so, adult male and female rats received (i.p.) temozolomide (25 mg/kg, 2 cycles, 5 days/cycle, 1 dose/day), cannabidiol (10 or 30 mg/kg, 3 doses within 24 h), fluoxetine (5–20 mg/kg, 3 doses within 24 h) alone and/or in combination. Antidepressant-like responses were scored under the stress of the forced-swim test. The results proved that all drugs induced antidepressant-like responses, although efficacy was related to sex and the dose administered. Moreover, the combination of fluoxetine with temozolomide was not different than the response induced by each drug separately. However, combining cannabidiol with temozolomide exhibited a sex-dependent antidepressant-like response solely observed in male rats, offering a potential therapeutic avenue to address depression in glioblastoma, where current antidepressants remain largely ineffective. Therefore, we propose that cannabidiol might be a good option to be administered in glioblastoma patients treated with temozolomide for improved affective-like responses, although not before its future validation in animal models of glioblastoma. Moreover, upcoming studies should center in personalizing the doses needed for this combinational approach to be also effective in females.

## 1 Introduction

Glioblastoma multiforme is one of the most lethal primary brain tumors, with a poor prognosis, frequent recurrence and limited treatment options (e.g., reviewed by Alifieris and Trafalis (2015) and more recently by Królikowska et al. (2025). Patients with glioblastoma often manifest prevalent and debilitating comorbidities like depression (e.g., Massie, 2004; Otto-Meyer et al., 2019; Mugge et al., 2020; Abazari et al., 2025), which are often worsened by chemotherapy and associated with poor quality of life and prognosis. Conventional antidepressant treatments show limited efficacy (e.g., Fisch, 2004; reviewed by Rabin et al. (2022)), emphasizing the need for novel therapeutic strategies compatible with oncologic regimens, ensuring minimal pharmacological interactions when tackling the neuropsychiatric burden associated with glioblastoma therapy.

The standard treatment for glioblastoma involves surgical resection, followed by concurrent radiotherapy and temozolomide chemotherapy (Angom et al., 2023; Jezierzański et al., 2024). Temozolomide is an alkylating agent that exerts cytotoxic effects through DNA methylation ultimately leading to DNA damage and apoptosis (Jezierzański et al., 2024), while also impairs hippocampal neurogenesis (Garthe et al., 2009; Nokia et al., 2012; Egeland et al., 2017; García-Cabrerizo et al., 2020). Prior preclinical research either suggested a link between temozolomide administration and the manifestation of depressive- and anxiety-like behaviors (e.g., Egeland et al., 2017; Pereira-Caixeta et al., 2018; Gan et al., 2019; Dey et al., 2020; Cabrera-Muñoz et al., 2023) or alternatively proposed the induction of antidepressant-like responses (Gálvez-Melero and García-Fuster, 2026). Overall, temozolomide’s depressant-vs. antidepressant-like profiles appeared to be cycle- and/or treatment-length-dependent and involving other mechanisms of action beyond impaired hippocampal neurogenesis (Gálvez-Melero and García-Fuster, 2026). The discrepancies in the neuropsychopharmacological responses of temozolomide might be somehow related to the emergence of resistance over the course of treatment.

In searching for more efficacious treatment strategies for glioblastoma that might help avoid therapeutic resistance, and temozolomide’s limited drug penetration across the blood-brain barrier, a recent approach has been to combine temozolomide with new therapeutic applications for existing drugs (reviewed by Chen and Alder (2025), Drappatz and Mantica (2025), Hsu et al. (2026)). In this context, cannabinoids have recently gained a lot of attention in cancer pharmacotherapy, not only for their use in palliative care but also for their suggested effects as antitumoral agents (e.g., Bukowska, 2024; Gómez-Lázaro et al., 2025; Javid et al., 2025). In fact, preclinical studies have suggested that cannabinoids, mainly cannabidiol and/or its combination with THC, can sensitize glioblastoma cells to the effects induced by temozolomide (recently reviewed by Javid et al. (2025)). In this context, efforts are already being made to translate these results into the clinic as several trials assess the efficacy of a 1:1 mixture of cannabidiol and THC in combination with temozolomide in patients with recurrent glioblastoma (Twelves et al., 2021; Sepúlveda et al., 2024; Bhaskaran et al., 2024). Thus, combination therapies, particularly cannabidiol with temozolomide exhibited additive or synergistic anti-tumor effects, capable of improving temozolomide’s resistance over the course of treatment (Bukowska, 2024; Gómez-Lázaro et al., 2025; Javid et al., 2025). For example, cannabidiol has been proven to enhance temozolomide cytotoxicity through TRPV2 activation (Nabissi et al., 2015). Moreover, cannabidiol inhibits human glioma by induction of lethal mitophagy through activating TRPV4 (Huang et al., 2021), thus leading the authors to propose that cannabidiol should be tested clinically for glioma, both alone and in combination with temozolomide.

Since cannabidiol has gained increasing interest due to its multifaceted anticancer properties and favorable safety profile (i.e., non-psychoactive with low toxicity and high tolerability; Bukowska, 2024; Gómez-Lázaro et al., 2025; Javid et al., 2025), and has shown a promising antidepressant-like profile in preclinical models and human studies (e.g., Guldager et al., 2024; Alexander et al., 2025; Miao et al., 2025), the present study aimed at evaluating the antidepressant-like profile induced by the combined therapy of cannabidiol and temozolomide, beyond its antitumoral properties, and focused on the potential treatment of glioblastoma-associated comorbidities. Moreover, fluoxetine, the goal standard treatment for depressive-like symptomatology will also be evaluated in combination with temozolomide, not only as a putative antidepressant-like control, but also given the potential application of antidepressant drugs in cancer treatment (reviewed by Zheng et al., 2023; Sun et al., 2025), and since recent studies have also suggested successful synergistic combinations of fluoxetine and temozolomide in the context of glioblastoma treatment (e.g., Liu et al., 2015; Song et al., 2015; Ma et al., 2016; Bi et al., 2021; reviewed by Kadasah et al., 2024) and/or affective-like behavior (e.g., Gan et al., 2019).

## 2 Materials and methods

### 2.1 Animals

**FIGURE 1:**
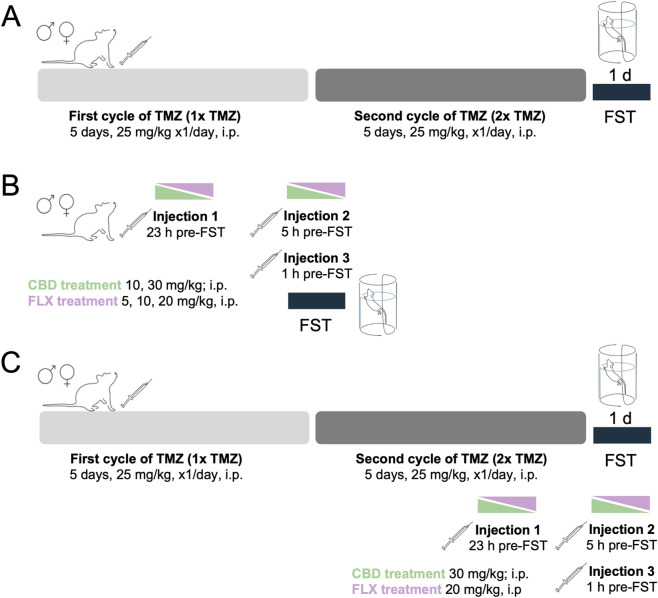
Experimental procedures. **(A)** Validating the antidepressant-like response of temozolomide (TMZ). TMZ was administered during two-cycles (2x TMZ, 10 days total with two resting days in between cycles, 25 mg/kg, x1/day, i. p.) in adult rats of both sexes. The forced-swim test (FST) was performed 1-day post-treatment. **(B)** Characterizing the dose-dependent antidepressant-like effects of cannabidiol (CBD) and fluoxetine (FLX). Prior to any pharmacological treatment, rats were forced to swim for 15 min (pre-test session). At the end of the swimming session, rats received three injections of CBD (10 or 30 mg/kg, i. p.), FLX (5, 10 or 20 mg/kg, i. p.) or the corresponding vehicle (Veh: 1:1.4 saline, 0.9% NaCl, and 1 mL/kg of DMSO) 23, 5 and 1 h before being exposed again to the FST for 5 min. **(C)** Studying changes in antidepressant-like efficacy by TMZ concomitant treatment. Rats were treated with TMZ for two cycles (25 mg/kg, i. p., 5 days/cycle, one dose/day, 10 days total with two resting days in between cycles). On the last day of treatment, rats received three injections of either CBD (30 mg/kg, i. p.), FLX (20 mg/kg, i. p) or the corresponding vehicle (Veh: 1:1.4 saline, 0.9% NaCl, and 1 mL/kg of DMSO) 23, 5 and 1 h prior to exposure to the FST for 5 min.

The present study, divided in three different objectives (Figure 1), utilized a total of 241 adult Sprague-Dawley rats (125 males, 116 females) bred in the animal facility at the University of the Balearic Islands. All procedures were revised and approved by the Local Bioethical Committee (SSBA 21/2024 AEXP; Conselleria Medi Ambient, Agricultura i Pesca, Direcció General Agricultura i Ramaderia, Govern de les Illes Balears) following ARRIVE guidelines (Percie du Sert et al., 2020) and EU Directive 2010/63/EU. Rats were separated at weaning in standard cages (2–4 rats/cage/sex) and housed in a controlled environment (22 °C, 70% of humidity, and a 12:12 h light/dark cycle, lights on at 8:00 a.m.) with access to food and water. Experimental groups for the different experiments were allocated and counterbalanced based on their basal performance (time spent (s) immobile, climbing or swimming) in the forced-swim test as previously described (e.g., García-Fuster and García-Sevilla, 2016; García-Cabrerizo et al., 2020; Gálvez-Melero and García-Fuster, 2026), to ensure a normal distribution and homogeneity of variance for all groups (see Supplementary Figure S1 for more experimental details). Moreover, this is relevant since circannual changes in the duration of the immobility response of rats has been shown for the forced-swim test (Abel, 1995), and thus, having a way of controlling among different batches of experiments becomes necessary. The estrous cycle phase was evaluated at the time of sacrifice in a subset of randomly selected female rats (see Supplementary Figure S2 for more experimental details and results). All efforts were directed towards minimizing the number of rats used, the number of procedures, which were always performed during the light-period, and their suffering.

### 2.2 Validating the antidepressant-like response of temozolomide

The first goal was centered in validating previous findings from our group reporting the antidepressant-like effects of a particular treatment paradigm of temozolomide in adult naïve rats of both sexes (Gálvez-Melero and García-Fuster, 2026). To do so, we used 58 rats (30 males and 28 females) run at two different time-points in separate batches that were divided in 2 experimental groups per sex and were treated with either temozolomide (25 mg/kg, i. p.; Merck, Sigma-Aldrich, PHR1437) or vehicle (1 mL/kg, i. p.) for 2 cycles (5 days/cycle, 1 dose/day, 10 days total with 2 resting days in between cycles) as shown in Figure 1A. This procedure therefore rendered the following experimental groups: Vehicle (Veh)-male (n = 15); 2x TMZ-male (n = 15); Veh-female (n = 14); 2x TMZ-female (n = 14). As mentioned, the selection of the dose and length of temozolomide treatment was based on our prior study determining the optimal conditions for temozolomide to induce an antidepressant-like response without affecting locomotor response in the open field or novelty-suppressed feeding test (Gálvez-Melero and García-Fuster, 2026), while also reducing basal neurogenic markers in the hippocampus (e.g., García-Cabrerizo et al., 2020; Gálvez-Melero and García-Fuster, 2026). Ensuring temozolomide at the doses tested did not affect locomotion was pertinent since activity is a confounding variable in the forced-swim test (see Gálvez-Melero and García-Fuster, 2026). Based on our prior results utilizing other behavioral tests and/or ensuring that locomotor response was not altered by the treatment conditions (Gálvez-Melero and García-Fuster, 2026), we concluded that the forced-swim test was the best great screening tool for evaluating temozolomide’s antidepressant-like responses.

Therefore, the antidepressant-like potential of temozolomide was scored under the stress of the forced-swim test, the goal standard screening tool for antidepressants in rodents (Slattery and Cryan, 2012), with predictive validity for its subsequent efficacy at the clinical level. To do so, we relied on standard procedures (Slattery and Cryan, 2012), adapted for our experimental conditions and validated for many different antidepressants during the last 10 years (see, for example, García-Fuster and García-Sevilla, 2016; Ledesma-Corvi et al., 2022; Gálvez-Melero and García-Fuster, 2026). As stated earlier, all rats were previously exposed to the pre-test to counterbalance the groups for immobility before treatment. Then, 1-day after the last treatment dose, rats from the first study (Figure 1A) were exposed to a 5-min forced-swim test session in which they were individually placed in water tanks (41 cm high x 32 cm diameter, 25 cm depth; temperature of 25 °C ± 1 °C). These sessions were videotaped to later evaluate the behavioral outcomes (time measured in a 300 s test); decreased immobility paired with increased escaping responses (i.e., climbing and/or swimming) as indicative of antidepressant-like efficacy. The analysis was done under blinded conditions using the software Behavioral Tracker (CA, United States).

### 2.3 Characterizing the dose-dependent antidepressant-like effects of cannabidiol and fluoxetine

For this aim (Figure 1B), we relied again on the forced-swim test to characterize the antidepressant-like responses induced by cannabidiol in comparison with the goal treatment standard fluoxetine in adult naïve rats of both sexes. Rats of each biological sex received 3 injections of cannabidiol (10 or 30 mg/kg, i. p.; purity ≥98%; THC Pharm, Germany), fluoxetine (5, 10 or 20 mg/kg, i. p.; PHR1394, Merck, Sigma-Aldrich, Burlington, MA, United States) or the corresponding vehicle (Veh: 1:1.4 proportion of saline, 0.9% NaCl, and 1 mL/kg of DMSO) 23, 5 and 1 h before being exposed again to the forced-swim test for 5 min (Figure 1B). This design for the cannabidiol experiment (CBD) utilized 57 adult rats (32 males and 25 females) divided in the following groups: Veh-male (n = 16); CBD-10-male (n = 7); CBD-30-male (n = 9); Veh-female (n = 8); CBD-10-female (n = 9); CBD-30-female (n = 8). Similarly, 63 adult rats (31 males and 32 females) were used for the fluoxetine experiment (FLX) as follows: Veh-male (n = 7); FLX-5-male (n = 7); FLX-10-male (n = 9); FLX-20-male (n = 8); Veh-female (n = 6); FLX-5-female (n = 9); FLX-10-female (n = 9); FLX-20-female (n = 8). The doses of cannabidiol and behavioral test under study were selected based on prior studies performed in rodents (e.g., Bis-Humbert et al., 2020; Gálvez-Melero et al., 2025), as were the ones for fluoxetine (i.e., Ledesma-Corvi et al., 2022) that have proved antidepressant-like efficacy in the forced-swim test without affecting locomotor response. Again, the 5-min sessions were videotaped to later measure the behavioral outcomes under blinded conditions using the software Behavioral Tracker (CA, United States). The aim of this experiment was to select an efficacious dose of each antidepressant (cannabidiol or fluoxetine) in the forced-swim test to later ascertain its potential pharmacological interaction with temozolomide.

### 2.4 Studying changes in antidepressant-like efficacy by temozolomide concomitant treatment

Finally, our last aim centered in studying the potential behavioral pharmacological interactions between the concomitant treatment with temozolomide and the selected antidepressants (cannabidiol, fluoxetine) in adult naïve rats of both sexes (i.e., temozolomide-vehicle, temozolomide-antidepressant for each sex). Each antidepressant interaction was evaluated in a separate cohort of rats and was performed on different days. Since the effects of temozolomide and cannabidiol or fluoxetine alone were characterized in the previous aims, in an attempt to reduce the number of rats used, the control groups were not repeated again, and thus in this last experiment all rats were treated with temozolomide and the variable of study was receiving or not the specific antidepressant (cannabidiol or fluoxetine). Briefly, rats were treated with temozolomide for 2 cycles (25 mg/kg, i. p., 5 days/cycle, 1 dose/day, 10 days total with 2 resting days in between cycles; Figure 1C). On the last day of temozolomide treatment, and after the last dose, rats were treated with 3 injections of cannabidiol (30 mg/kg, i. p.), fluoxetine (20 mg/kg, i. p) or the corresponding vehicle (1:1.4 proportion of saline, 0.9% NaCl and 1 mL/kg of DMSO) 23, 5 and 1 h prior to exposure to the forced-swim test for 5 min (Figure 1C). The design for cannabidiol (CBD) utilized 33 adult rats (17 males and 16 females) that were divided in the following groups: TMZ-Veh-male (n = 8); TMZ-CBD-male (n = 9); TMZ-Veh-female (n = 7); TMZ-CBD-female (n = 9). Similarly, 30 adult rats (15 males and 15 females) were used for the fluoxetine experiment (FLX) as follows: TMZ-Veh-male (n = 7); TMZ-FLX-male (n = 8); TMZ-Veh-female (n = 7); TMZ-FLX-female (n = 8). The behavioral interactions at the antidepressant-like efficacy level were evaluated in the 5-min sessions that were videotaped under blinded conditions using the software Behavioral Tracker (CA, United States).

### 2.5 Statistical analysis

Data analysis and plotting was done with GraphPad Prism, Version 10 (GraphPad Software, CA, United States). Individual symbols are shown for each rat and the mean value ±standard error of the mean (SEM) is shown for each treatment group. All data was analyzed including sex as a biological variable through two-way ANOVAs (independent variables: biological sex and treatment; see Supplementary Table S1 for statistical details) and *post hoc* comparisons when appropriate. On the right panel of each figure, we showed a combined analysis including rats of both sexes (mixed-sex cohort) through one-way ANOVAs or unpaired two-tail Student’s *t*-tests as appropriate. The level of significance was set at *p* ≤ 0.05. Data supporting the present findings will be available upon reasonable request to the corresponding author.

## 3 Results

### 3.1 Temozolomide induced antidepressant-like efficacy

**FIGURE 2:**
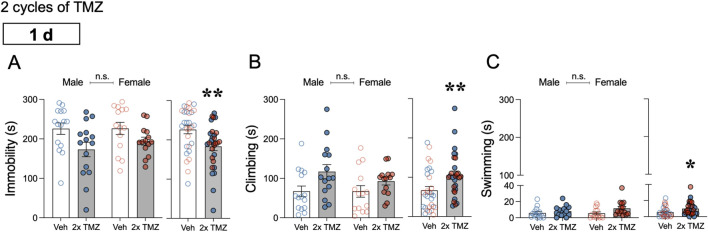
Antidepressant-like effects induced by temozolomide (TMZ) under the stress of the forced-swim test (FST) in adult rats of both sexes. Changes in **(A)** immobility (s), **(B)** climbing (s), or **(C)** swimming (s) induced by two TMZ cycles (2x TMZ, 10 days total with two resting days in between them, 25 mg/kg, x1/day, i. p.) or Veh as measured 1-day post-treatment under the stress of the FST in male and female adult rats. Groups of treatment: Vehicle (Veh)-male (n = 15); 2x TMZ-male (n = 15); Veh-female (n = 14); 2x TMZ-female (n = 14). Columns represent mean ± SEM of the each of the defined measurements. Individual values are shown for each rat. Data is shown separately for each biological sex and as a mixed-sex cohort group (right panels). Two-way ANOVAs analyses (independent variables: biological sex and treatment) are shown in Supplementary Table S1. Rats of both sexes were combined to assess the overall effect of TMZ treatment through Student *t*-tests (**p < 0.01 when comparing 2x TMZ vs. Veh-treated rats).

The results showed that ×2 cycles of temozolomide treatment (10 days) induced an antidepressant-like response when measured in the forced-swim test 1-day post-treatment in adult naïve rats (Figure 2; see Supplementary Table S1 for a detailed statistical analysis). Treatment efficacy was observed as an overall effect for immobility, climbing and swimming (Supplementary Table S1), which was also present when combining rats of both sexes (mixed-sex cohort). Particularly, ×2 cycles of temozolomide induced a significant reduction in immobility as observed 1-day post-treatment in a mixed-sex cohort of adult rats (−43 ± 15 s, *t* = 2.89, *df* = 56, **p = 0.006 vs. vehicle-treated rats; Figure 2A). This reduced immobility aligned with increased climbing (+38 ± 14 s, *t* = 2.69, *df* = 56, **p = 0.009 vs. vehicle-treated rats; Figure 2B) and swimming (+4 ± 2 s, *t* = 2.23, *df* = 56, *p = 0.030 vs. vehicle-treated rats; Figure 2C) behaviors, indicative of antidepressant-like efficacy.

### 3.2 Sex- and dose-dependent antidepressant-like effects of cannabidiol and fluoxetine

**FIGURE 3:**
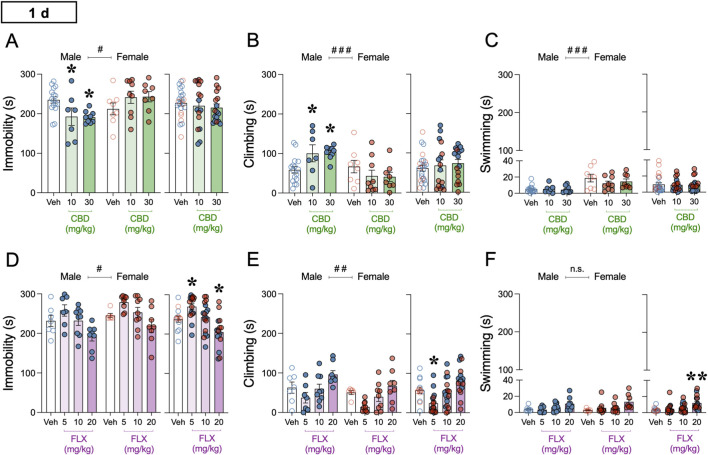
**S**ex- and dose-dependent antidepressant-like effects of cannabidiol (CBD) and fluoxetine (FLX). Changes in **(A,D)** immobility (s), **(B,E)** climbing (s), or **(C,F)** swimming (s) induced by 3 injections of CBD (10 or 30 mg/kg, i. p.), FLX (5, 10 or 20 mg/kg, i. p.) or their corresponding vehicle (Veh: 1:1.4 saline, 0.9% NaCl, and 1 mL/kg of DMSO), as received 23, 5 and 1 h before rats of both sexes were exposed to the FST. Groups of treatment: **(A–C)** Veh-male (n = 16); CBD-10-male (n = 7); CBD-30-male (n = 9); Veh-female (n = 8); CBD-10-female (n = 9); CBD-30-female (n = 8). **(D–F)** Veh-male (n = 7); FLX-5-male (n = 7); FLX-10-male (n = 9); FLX-20-male (n = 8); Veh-female (n = 6); FLX-5-female (n = 9); FLX-10-female (n = 9); FLX-20-female (n = 8). Columns represent mean ± SEM of the each of the defined measurements. Individual values are shown for each rat. Data is shown separately for each biological sex and as a mixed-sex cohort group (right panels). Two-way ANOVAs analyses (independent variables: biological sex and treatment) are shown in Supplementary Table S1 (###*p* < 0.001, ##*p* < 0.01 and #*p* < 0.05 when comparing male vs. female rats). Rats of both sexes were combined to assess the overall effect of treatment through one-way ANOVAs followed by *post hoc* analysis when appropriate. **p < 0.01 and *p < 0.05 vs. Veh-treated rats.

The results showed sex-specific and dose-dependent antidepressant-like responses for cannabidiol and/or fluoxetine in adult naïve rats under the stress of the forced-swim test (Figure 3). In particular, the results for the experiment with cannabidiol showed significant effects of biological sex for all behavioral features analyzed in the test (Figures 3A–C; overall effects of sex represented by the symbol # and as reported in its figure legend), and significant interactions between biological sex x treatment when evaluating immobility (Figure 3A) and climbing (Figure 3B), as detailed in the Supplementary Table S1. Dunnett’s multiple comparisons test showed a significant reduction in immobility for male rats at both doses tested (10 mg/kg: −42 ± 18 s, *p = 0.039; 30 mg/kg: −44 ± 16 s, *p = 0.018; Figure 3A), paired with an increase in climbing 10 mg/kg: +42 ± 17 s, *p = 0.039; 30 mg/kg: −44 ± 16 s, *p = 0.016; Figure 3B), when compared to vehicle-treated rats. These antidepressant-like effects were sex-specific, since they were not observed in female rats, and got dissipated when combining all rats in a mixed-sex cohort group (Figures 3A,B). Cannabidiol induced antidepressant-like efficacy through increasing climbing, since no statistical differences were observed for swimming behavior (Supplementary Table S1; Figure 3C).

As for the experiment with fluoxetine, the results showed that although biological sex accounted for some differences in the responses observed for immobility (Figure 3D) and climbing (Figure 3E), the main results presented clear effects of treatment for all behavioral features analyzed that were not affected by biological sex (i.e., lack of sex × treatment interactions; Supplementary Table S1). Particularly, fluoxetine induced different responses in immobility that were dose-dependent when analyzing all rats as a mixed-sex cohort through a one-way ANOVA (F_3, 59_ = 9.29, *p* < 0.001; right panel, Figure 3D); while the dose of 5 mg/kg seemed to be deleterious (+32 ± 13 s, *p = 0.044), the highest dose tested of 20 mg/kg showed signs of antidepressant-like efficacy (−33 ± 13 s, *p = 0.037) when compared to vehicle-treated rats (Figure 3D). Interestingly, the deleterious increase in immobility induced by the dose of 5 mg/kg of fluoxetine combined with a decrease in climbing (F_3, 59_ = 8.29, *p* < 0.001; right panel, Dunnett’s *post hoc*: 33 ± 12 s, *p = 0.024; Figure 3E). Contrarily, the beneficial antidepressant-like effect induced by the dose of 20 mg/kg of fluoxetine paired with an increase in swimming behavior (F_3, 59_ = 5.81, *p* = 0.002; right panel, Dunnett’s *post hoc*: +8 ± 2 s, **p = 0.001; Figure 3F).

### 3.3 Antidepressant-like efficacy for cannabidiol when combined with temozolomide

**FIGURE 4:**
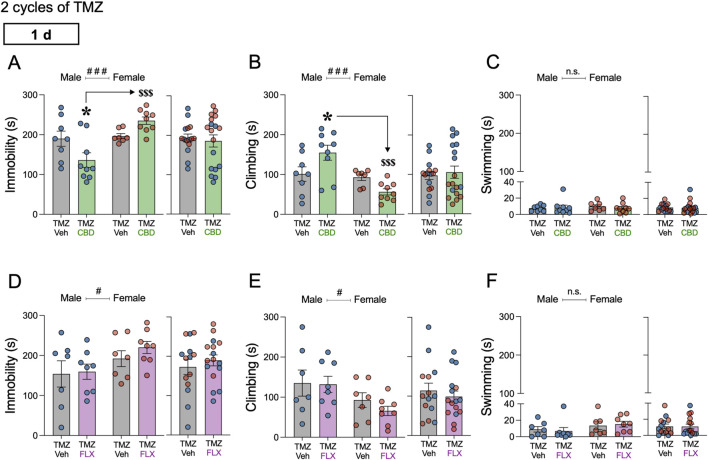
Studying changes in antidepressant-like efficacy by a temozolomide (TMZ) concomitant treatment. Changes in **(A,D)** immobility (s), **(B,E)** climbing (s), or **(C,F)** swimming (s) induced by 2 cycles of TMZ (25 mg/kg, i. p., 5 days/cycle, 1 dose/day, 10 days total with 2 resting days in between cycles) combined with 3 injections of either CBD (30 mg/kg, i. p.), FLX (20 mg/kg, i. p) or the corresponding vehicle (Veh: 1:1.4 saline, 0.9% NaCl, and 1 mL/kg of DMSO) 23, 5 and 1 h prior to exposure to the FST for 5 min. Groups of treatment: TMZ-Veh-male (n = 8); TMZ-CBD-male (n = 9); TMZ-Veh-female (n = 7); TMZ-CBD-female (n = 9). TMZ-Veh-male (n = 7); TMZ-FLX-male (n = 8); TMZ-Veh-female (n = 7); TMZ-FLX-female (n = 8). Columns represent mean ± SEM of the each of the defined measurements. Individual values are shown for each rat. Data is shown separately for each biological sex and as a mixed-sex cohort group (right panels). Two-way ANOVAs analyses (independent variables: biological sex and treatment) are shown in Supplementary Table S1 (###*p* < 0.001 and #*p* < 0.05 when comparing male vs. female rats). No significant effects were reported when rats of both sexes were combined to assess the overall effect of treatment through Student *t*-tests.

For studying the potential changes in antidepressant-like response induced by a prior and/or concomitant treatment with temozolomide, we selected, based on the prior study, the dose of 30 mg/kg of cannabidiol and 20 mg/kg of fluoxetine. The behavioral results for cannabidiol showed significant effects of biological sex for immobility (Figure 4A) and climbing (Figure 4; overall impact of sex represented by the symbol # and as reported in its figure legend), and significant interactions between biological sex x treatment when evaluating immobility (Figure 4A) and climbing (Figure 4B), as detailed in the Supplementary Table S1. *Post-hoc* multiple comparisons test showed that the group treated concomitantly with temozolomide and cannabidiol showed reduced immobility (−54 ± 21 s, *p = 0.038; Figure 4A) and increased climbing (−54 ± 20 s, *p = 0.035; Figure 4B) as compared to the respective temozolomide-vehicle-treated group. Moreover, a significant difference emerged when comparing male and female rats treated with the combination of temozolomide and cannabidiol; male rats showed significantly lower immobility rates as compared to females (−99 ± 20 s, $$$*p* < 0.001; Figure 4A), paired with increased climbing (+98 ± 20 s, $$$*p* < 0.001; Figure 4B). These results dissipated when combining all rats in a mixed-sex cohort group (Figures 4A,B). Again, cannabidiol induced these sex-specific effects with temozolomide through modulating climbing as no statistical differences were observed for swimming behavior (Supplementary Table S1; Figure 4C).

As for the experiment with fluoxetine, the results showed significant overall effects of biological sex for immobility (Figure 4D) and climbing (Figure 4E; Supplementary Table S1), but no significant outcomes induced by treatment and/or biological sex × treatment interactions for all behavioral measurements analyzed (Figures 4D–F; Supplementary Table S1). As hypothesized for cannabidiol, since temozolomide alone and/or fluoxetine alone exerted antidepressant-like efficacy by decreasing immobility in the forced-swim test, the response of fluoxetine in the presence of temozolomide probably represented a maximized test effect (TMZ-FLX vs. TMZ-Veh; Figures 4D–F).

## 4 Discussion

This study aimed at evaluating the behavioral pharmacological interaction of temozolomide with two antidepressants (i.e., cannabidiol and fluoxetine) in adult naïve rats while incorporating sex as a biological variable. The main results observed in the forced-swim test showed that: (1) temozolomide induced an antidepressant-like response following a 2-cycle treatment in a mixed-sex cohort of adult rats; (2) cannabidiol exerted sex- and dose-dependent antidepressant-like efficacy, exclusively observed in male rats; (3) fluoxetine induced either deleterious or antidepressant-like effects in a dose-dependent manner in a mixed-sex cohort of adult rats; and (4) when these antidepressants were tested in rats previously treated with temozolomide, the combination with cannabidiol induced sex-specific effects, but exclusively in male rats, while its combination with fluoxetine did not alter the previously antidepressant-like effects of each drug individually. Thus, we provide information regarding the beneficial effects of a combinational therapy of cannabidiol and temozolomide aiming at inducing antidepressant-like effects for eventually treating the most associated comorbidity of glioblastoma, depression. While this experimental design in naïve rats allowed us to evaluate the behavioral effects of the treatment under controlled conditions and minimize potential confounding factors associated with tumor progression, it did not fully reproduce the complex pathophysiological environment of glioblastoma. Therefore, the behavioral responses observed in naïve rats may differ from those occurring in tumor-bearing subjects, and thus, the present findings should be interpreted with caution when considering their clinical relevance to glioblastoma patients.

The first part of this study aimed at validating the antidepressant-like potential of temozolomide under the stress of the forced-swim test. Our results proved that 2 cycles of temozolomide administered during 2 consecutive weeks induced antidepressant-like efficacy in a mixed-sex cohort of adult rats, confirming our prior recent data (Gálvez-Melero and García-Fuster, 2026), which in line with former studies suggested cycle- and/or length-dependent treatment effects in terms of temozolomide’s antidepressant-vs. depressant- and/or anxiogenic-like profile (e.g., Egeland et al., 2017; Pereira-Caixeta et al., 2018; Gan et al., 2019; Dey et al., 2020; Cabrera-Muñoz et al., 2023). Therefore, any mood changes associated with temozolomide treatment for glioblastoma might be associated with longer treatment paradigms together with the concomitant manifestation of depressive-like behaviors, since shorter temozolomide treatments could be beneficial for such comorbidities (present data and Gálvez-Melero and García-Fuster, 2026).

The second part of the study aimed at ascertaining the proper doses of cannabidiol and fluoxetine needed to induce antidepressant-like responses in our experimental conditions in adult naïve rats while exposed to the stress of the forced-swim test. Cannabidiol induced sex- and dose-dependent antidepressant-like responses exclusively in male rats by decreasing immobility under the stress of the forced-swim test, while increasing climbing behavior. Climbing is typically associated with antidepressants known to mediate their response through enhancements in noradrenaline-mediated signaling (Detke et al., 1995), as well as in relation to cannabidiol (Bis-Humbert et al., 2020; Ledesma-Corvi et al., 2022; Gálvez-Melero et al., 2025). These results align with prior studies demonstrating the preclinical efficacy of cannabidiol in male rats while lacking efficacy in females (e.g., Bis-Humbert et al., 2020; Silote et al., 2021; Ledesma-Corvi et al., 2022; Gálvez-Melero et al., 2025). As for fluoxetine, the effects induced in the forced-swim test were observed for rats of both sexes (mixed-sex cohort). While a lower dose (5 mg/kg) induced a deleterious effect, the higher dose tested (20 mg/kg) exerted antidepressant-like efficacy, in line with prior studies showing efficacy at 10 mg/kg (e.g., Ledesma-Corvi et al., 2022). Interestingly, and contrarily to what was observed with cannabidiol, the beneficial effects of fluoxetine were manifested as increases in swimming behavior, as previously described for serotonin reuptake inhibitors (Detke et al., 1995). From this study, we selected the dose of 30 mg/kg of cannabidiol and the dose of 20 mg/kg of fluoxetine, shown to not affect locomotor responses (Bis-Humbert et al., 2020; 2021; Ledesma-Corvi et al., 2022), to be combined with temozolomide and ascertain potential drug interactions at the behavioral level.

Finally, the last study aimed at providing information regarding the potential beneficial effects of a combinational therapy of temozolomide with an antidepressant in the context of finding better options for the future treatment of glioblastoma associated comorbidities, other than centering in the more broadly explored synergic anti-tumoral effects of these treatment combinations (Bukowska, 2024; Gómez-Lázaro et al., 2025; Javid et al., 2025). The present results proved that the combination of temozolomide with cannabidiol induced sex-specific effects in the forced-swim test, but exclusively in male rats, while its combination with fluoxetine did not alter the previously antidepressant-like effects of each drug individually. Therefore, combination therapies such as cannabidiol and temozolomide not only exhibited additive or synergistic anti-tumor effects (Bukowska, 2024; Gómez-Lázaro et al., 2025; Javid et al., 2025) but also induced antidepressant-like responses in the forced-swim test. These results proved a mixture of drugs with dual efficacy and great relevance in the context of finding novel options for the future treatment of depression comorbidity in glioblastoma patients. Unfortunately, these beneficial effects were exclusively observed in male naïve rats, thus suggesting the future need for more personalized studies centered in females as well as in rodent models of glioblastoma to fully characterize their responses. On the other hand, fluoxetine proved antidepressant-like efficacy alone, but lacked a combined effect with temozolomide, contrarily to recent studies suggesting successful synergistic combinations of fluoxetine and temozolomide in the context of glioblastoma treatment (e.g., Liu et al., 2015; Song et al., 2015; Ma et al., 2016; Bi et al., 2021; reviewed by Kadasah et al., 2024) and/or affective-like behavior (e.g., Gan et al., 2019).

The present study is limited to the experimental conditions tested and should be further explored in the future. For example, the conclusions extracted for naïve rats in response to a stressor in the forced-swim test should be further validated with additional behavioral tests that also score antidepressant-like responses. Moreover, studies using rodent models of glioblastoma will be necessary to determine whether the observed antidepressant-like effects are maintained under tumor-associated conditions and to better establish the translational significance of our findings. Finally, it would be of great interest to evaluate the molecular signatures that differentially cannabidiol’s vs. fluoxetine’s response when combined with temozolomide, as well as to better understand the observed sex differences. In this context, and as further discussed in one of our prior studies including the antidepressant-like characterization of cannabidiol and fluoxetine in rats of both sexes (see Ledesma-Corvi et al., 2022), there are several possible contributing factors to the differences in efficacy observed for each drug and biological sex (i.e., sex hormone influences, differences in serotonergic or endocannabinoid signaling, pharmacokinetic variation, and/or sex-specific stress responses for each drug). Future studies will center in evaluating specific markers of antidepressant-like response such as BDNF and hippocampal neurogenesis (e.g., Gálvez-Melero et al., 2025) to evaluate if they could be mediating the sex-specific effects observed when combining cannabidiol and temozolomide in male rats.

## 5 Conclusion

In the context of glioblastoma treatment with temozolomide and with comorbid psychopathology requiring the use of an antidepressant treatment, our results propose the improved efficacy of cannabidiol in the forced-swim test, as compared to the goal standard fluoxetine. Similarly to prior results with cannabidiol, these beneficial effects were exclusively observed in male rodents, thus reinforcing the need for future studies ascertaining the personalized doses needed for females, as well as their future validation in rodent models of glioblastoma.

## Data Availability

The raw data supporting the conclusions of this article will be made available by the authors, without undue reservation.
