# Supplementary material for: Antidepressant-like effects of cannabidiol when combined with temozolomide in adult rats
Source: Front Pharmacol. 2026 Jun 29;17:1873308. doi: 10.3389/fphar.2026.1873308 (PMC13357983; doi:10.3389/fphar.2026.1873308)
Supplement: Supplementary file 1 [file Supplementaryfile1.docx]

**Supplementary Materials**

**Fig. S1.** **Basal performance in the forced-swim test prior to any drug treatment.** Data represents the time spent (s) immobile, climbing or swimming in the forced-swim test as performed prior to any drug treatment for the rats included in the experimental detailed Fig. 1A and Fig. 1C (male: n = 47, female: n = 45). To do so, all rats were individually placed for 15-min (pre-test session) in water tanks (41 cm high x 32 cm diameter, 25 cm depth; temperature of 25 ± 1 °C), followed by a 5-min test session that was performed the next day while videotaped. Videos were blindly scored (Behavioral Tracker software, CA, USA) to establish individual basal levels of immobility (i.e., lack of movement except the one required to keep the rat’s nose above the water level) vs. active behaviors (climbing or swimming). The potential basal sex differences in the response of rats in this behavioral test were assessed through Student *t*-tests, which showed no statistical differences for any of the measurements (i.e., immobility: *t* = 1.92, *df* = 90, *p* = 0.058; climbing: *t* = 1.95, *df* = 91, *p* = 0.054; swimming: *t* = 1.86, *df* = 91, *p* = 0.066). These basal values were used to counterbalance rats and allocate them in different treatment groups as detailed in Fig. 1A and Fig. 1C.

**Fig. S2.** **Estrous cycle phases of female rats.** The specific stages of the estrous cycle were monitored at the time of sacrifice for female rats included in the experiment detailed in Fig. 1A (28 females) and 1C (31 females). The experimental procedure was performed as follows; we collected vaginal fluid with cotton swabs dipped in 0.9% saline and spread it on a slide that was saved at -80 °C until further analysis. The estrous cycle phases were monitored under a light microscope following a cresyl violet staining. Data represents a chart with the number of female rats in each phase observed. Out of the female rats evaluated, the results showed that most rats were transitioning between diestrus (10%) to proestrus (80%) or proestrus to estrus (10%) stages, with no rats found in metestrus (0%). Thus, note that most female rats were either transitioning either from diestrus to proestrus or proestrus to estrus, or were in estrus phase, while no rats were in metestrus.

**Table. S1.** Two-way ANOVAs analyses (independent variables: Biological Sex and Treatment), both including F (DFn, DFd) and *p* values, for data represented in Figures 2-4. Color-shadow boxes represent statistically significant comparisons.
